# Integrated delivery of family planning and childhood immunization services: a qualitative study of factors influencing service responsiveness in Malawi

**DOI:** 10.1093/heapol/czac048

**Published:** 2022-06-17

**Authors:** Jessie K Hamon, Misozi Kambanje, Shannon Pryor, Alice S Kaponda, Erick Mwale, Susannah H Mayhew, Jayne Webster, Helen E D Burchett

**Affiliations:** Department of Disease Control, LSHTM, Keppel Street, London WC1E 7HT, UK; Save the Children, Blantyre Field Office, Mandala, Private/Bag 254, Blantyre, Malawi; Save the Children, 501 Kings Highway East, Suite 400, Fairfield, CT 06825, USA; Save the Children, Blantyre Field Office, Mandala, Private/Bag 254, Blantyre, Malawi; Save the Children, Blantyre Field Office, Mandala, Private/Bag 254, Blantyre, Malawi; Department of Global Health and Development, LSHTM, 15-17 Tavistock Pl, London WC1H 9SH, UK; Department of Disease Control, LSHTM, Keppel Street, London WC1E 7HT, UK; Department of Public Health, Environments and Society, LSHTM, 15-17 Tavistock Pl, London WC1H 9SH, UK

**Keywords:** Family planning, immunization, integration, service delivery, responsiveness, hardware, software, Malawi

## Abstract

Evidence from several countries in sub-Saharan Africa suggests that the integration of family planning (FP) with childhood immunization services can help reduce the unmet need for FP among postpartum women without undermining the uptake of immunizations. However, the quality and responsiveness of FP services that are integrated with childhood immunizations remain understudied. A qualitative study was conducted in two districts of Malawi, which examined the factors influencing the responsiveness of FP services that were integrated with childhood immunizations in monthly public outreach clinics. Semi-structured interviews with clients (*n* = 23) and FP providers (*n* = 10) and a clinic audit were carried out in six clinics. Hardware (material) and software (relational) factors influencing service responsiveness were identified through thematic and framework analyses of interview transcripts, and clinic characteristics were summarized from the audit data to contextualize the qualitative findings. Overall, 13 factors were found to influence service responsiveness in terms of the ease of access, choice of provider, environment, service continuity, confidentiality, communication, dignity and FP counselling afforded to clients. Among these factors, hardware deficiencies, including the absence of a dedicated building for the provision of FP services and the lack of FP commodities in clinics, were perceived to negatively affect service responsiveness. Crucially, the providers’ use of their agency to alter the delivery of services was found to mitigate the negative effects of some hardware deficits on the ease of access, choice of provider, environment and confidentiality experienced by clients. This study contributes to an emerging recognition that providers can offset the effect of hardware deficiencies when services are integrated if they are afforded sufficient flexibility to make independent decisions. Consideration of software elements in the design and delivery of FP services that are integrated with childhood immunizations is therefore critical to optimize the responsiveness of these services.

Key messages

- According to FP clients and providers, hardware deficiencies, such as the absence of a suitable building, inappropriate service structure and insufficient commodities, undermine the responsiveness experienced by clients utilizing FP services that are integrated with childhood immunizations in routine outreach clinics. This is concerning because the responsiveness of services is critical to the uptake and continuous use of contraceptives given the sensitive and repeat nature of FP services.
- Crucially, FP providers can mitigate the negative effect of some hardware deficits by applying their agency to alter the delivery of services so long as the hardware in question does not relate to providers themselves (e.g. staffing shortages or knowledge gaps). Consideration of software elements such as the providers’ agency in the design and delivery of integrated FP services is therefore pivotal to the responsiveness of services.
- Programme designers and implementers looking to improve the responsiveness of FP services that are integrated with childhood immunizations should not only create the conditions under which providers can apply their agency to pragmatically deliver services but should also provide the tools for them to adapt services in a way that ensures clients’ legitimate expectations are met. However, it is equally paramount to address hardware gaps so that providers’ efforts are not dominated by attempts to mitigate the negative effects of these deficiencies.

## Introduction

The integration of family planning (FP) with childhood immunization services is recognized as a promising approach to reduce the unmet need for FP among postpartum women, prevent unintended pregnancies and facilitate healthy birth spacing by creating repeat opportunities for FP services to reach underserved women (High Impact Practices in Family Planning (HIP), 2021). Although FP is more commonly integrated with HIV services in sub-Saharan Africa, there is mounting evidence from several countries to suggest that the integration of FP with childhood immunizations can increase FP without undermining the uptake of immunizations (Huntington and Aplogan, 1994; Cooper *et al.*, 2015; 2020; Dulli *et al.*, 2016; Nelson *et al.*, 2019). However, the experiential quality and responsiveness of FP services that are integrated with childhood immunizations remain understudied.

Service responsiveness is conceptualized as the extent to which an individual’s interaction with a specific health service fulfils a set of universally accepted ethical principles and non-clinical service standards (de Silva, 2000; Murray and Frenk, 2000; World Health Organization, 2000; Darby *et al.*, 2003; Khan *et al.*, 2021). According to the literature on the responsiveness and quality of FP services (Perera *et al.*, 2011; 2012a; 2012b; RamaRao and Jain, 2016; Tessema *et al.*, 2016; 2017; Jain and Hardee, 2018), both structural and behavioural domains of responsiveness are relevant to the integrated delivery of FP services. Structural domains include the ease of access, choice of provider, environment and the service continuity experienced by clients, whereas behavioural domains include the confidentiality, communication, dignity and the counselling afforded to clients. Although these domains are not critical to the clinical quality of FP services, they shape clients’ perceptions of health services and determine their willingness to repeat their use (Hanefeld *et al.*, 2017), which is central to FP services given their sensitive and often repeat nature (e.g. for users of short-acting contraceptives) (Bossyns *et al.*, 2002; Harris *et al.*, 2016; Fruhauf *et al.*, 2018; Jain and Hardee, 2018; Senderowicz, 2020).

In 2019, a case study of the responsiveness of FP services that were integrated with childhood immunizations was carried out in Malawi. Findings from the first part of this case study demonstrated that when delivered with childhood immunizations in routine outreach clinics, FP services can be responsive in terms of dignity, service continuity, communication and access to services afforded to clients (Hamon *et al.*, 2022). It also revealed that the choice of provider, environment and confidentiality that clients experienced in this context was less than ideal. However, the factors influencing the responsiveness of integrated FP services remain unclear.

This article reports findings from the second part of the case study, which involved a qualitative investigation of clients’ and providers’ views and experiences and aimed to identify the factors influencing the responsiveness of FP services that were integrated with childhood immunizations. A secondary aim was to ascertain the effect of people’s values, beliefs and relations on service responsiveness, as few studies on the integration of FP services have examined these (Phiri *et al.*, 2016; Mutisya *et al.*, 2019; Mayhew *et al.*, 2020). In fact, research to date has almost exclusively focused on the influence of material factors such as the infrastructure and resources (staff and commodities) at the point of care (Huntington and Aplogan, 1994; FHI 360, 2012; Cooper *et al.*, 2015; 2020; Dulli *et al.*, 2016; Nelson *et al.*, 2019; Sheahan *et al.*, 2021).

## Methods

### Study setting

The study was conducted in non-static public outreach clinics delivering the Expanded Programme on Immunization, in which FP services were integrated with childhood immunizations and growth monitoring services. Although these clinics were organized and provided by administrators and health workers operating in the public sector, the design and monitoring of the integration of FP services into these clinics were supported by a non-governmental organization (NGO).

The clinics included in this study were carried out during a single day each month in several rural communities across Malawi’s Blantyre and Thyolo districts where childhood immunization rates, modern contraceptive use rates and the unmet need for FP among married women were relatively similar to national averages (Table 1) (National Statistical Office (NSO) [Malawi] and ICF, 2017).

**Table 1.** National and district FP and immunization rates

	Blantyre district (%)	Thyolo district (%)	National average (%)
Rate of modern contraceptive use among married women age 15–49	60.3	58.7	58.1
Rate of unmet need for FP among married women age 15–49	18.7	18.9	18.7
Rate of children 12–23 months with all basic vaccinations	63.1	82.4	75.8
Rate of children 12–23 months with 3rd dose of DPT-HepB-Hib vaccine	85.7	91.4	93.0

In the studied clinics, services were organized using a standardized client flow (Hamon *et al.*, 2020). At the start of each clinic day, a group health talk was held, which covered topics such as child health and the benefits of FP and immunizations. Clients were then screened, and growth monitoring and childhood immunizations were carried out. Subsequently, women who were interested in FP services were counselled, short-acting contraceptives were offered and referrals to the nearest health centre were given to women seeking long-acting reversible contraceptives (LARCs). This client flow was designed to function with a minimum of four health surveillance assistants (HSAs) and support from community volunteers. In Malawi, HSAs are paid community health workers who provide health promotion and prevention services through health centres and community outreach activities (Kok *et al.*, 2020). Typically, they possess a secondary school level education and receive 12 weeks of pre-service education (Nyirenda *et al.*, 2014).

### Empirical data collection and analysis

Empirical data were collected between June and July 2019 by two trained local interviewers who were led by an experienced research coordinator and supervised by the lead researcher. Ethics approval was obtained for this study from the National Committee on Research in the Social Sciences and Humanities in Malawi and from the London School of Hygiene & Tropical Medicine ethics committee in the UK. Written informed consent was obtained from all respondents prior to their involvement in the study.

Only routine outreach clinics where FP services were integrated with childhood immunizations for at least 12 months were considered for inclusion in the study. Among the 16 clinics that met this inclusion criterion across the two districts, six were selected (three in each district). Sites were selected based on the NGO’s routine monitoring data and impressions of the clinics’ functioning during the previous 12 months to ensure that a range of clinics were included in the study. Indicators considered in the selection of sites were (1) the clinics’ average fulfilment of staffing standards (four HSAs or more), (2) the clinics’ average FP client load and (3) the level of involvement (high/low) from community members in the clinics as reported by the NGO team.

In each of the selected clinics, semi-structured interviews (SSIs) were conducted with clients and their FP providers. A convenience sampling approach was used to recruit all providers who delivered FP services in the six clinics on the day of the interviews and to recruit clients exiting the clinics, with the aim of interviewing four clients and one provider per clinic. Eligible clients were 18 years or older, had a child under the age of three years and sought both FP and childhood immunization services on the day of the interview. Clients who reported having a sick child or who had already been interviewed for other parts of the study were excluded.

The interview guides used to conduct the SSIs combined scripted open-ended and probing questions to facilitate discussion between the interviewer and respondent. The guide used to interview clients focused on their experiences receiving FP services in the outreach clinics, and the changes that they felt were needed to improve these experiences. Similarly, the guide used to interview providers focused on their experiences delivering FP services in outreach clinics, the improvements they felt were needed and the factors they believed influenced the providers’ and clinics’ ability to meet clients’ needs. Clients and providers were also prompted to share their thoughts on the integration of FP services with childhood immunizations. Additionally, clients were asked to explain which responsiveness domains they felt were most and least important, and providers were asked what they believed was most and least important to their clients. Both interview guides were piloted in two clinics to determine the suitability of the language and questions they contained. All interviews were conducted in either Chichewa or English, and detailed interview logs were kept by the interviewers to facilitate reflexivity during the data collection.

Additionally, an audit was carried out in each clinic to contextualize findings from the SSIs. The audit was completed by a trained data collector using a structured questionnaire with support from an HSA working in each clinic. The questionnaire included questions based on the WHO’s Service Availability and Readiness Assessment and the Quick Investigation of Quality tool developed by MEASURE Evaluation (World Health Organization, 2015; MEASURE Evaluation, 2016). It captured information on the clinic’s infrastructure, staffing and the availability of contraceptive and immunization commodities on the day of the SSIs. As with the SSI guides, the audit questionnaire was piloted in two clinics prior to its use in the studied clinics.

Following each data collection day, a debriefing session was held, in which the interviewers shared their impressions of the SSIs and the interviewer–respondent dynamics they experienced. Notes taken by the team coordinator and the lead researcher during these sessions helped ensure that emerging themes were carefully explored during subsequent interviews.

Audio recordings from the SSIs were transcribed *verbatim*, translated into English and imported into Nvivo 12 for coding and analysis by the lead researcher. Upon import, quotes were anonymized; however, the number assigned to each clinic during data collection and the type of respondent (client or provider) were retained to facilitate analyses. Thematic and framework analyses of the SSI transcripts were performed based on the principles of constructivism. First, the data were coded deductively along the eight responsiveness domains to identify the themes and sub-themes pertaining to each domain. Second, the clients’ and providers’ responses within each theme were compared to ascertain how they aligned/diverged. Third, dominant themes were scrutinized to identify the key factors believed by respondents to influence service responsiveness. Fourth, the factors were classified according to whether they were ‘hardware’ (material) or ‘software’ (relational) elements of the health system. As defined by Sheikh et al., hardware referred to the tangible elements of the health system, such as the resources, structures and forms of service delivery, whereas software included the attitudes, values, practices and power dynamics that defined the relationships between system actors, elements and contexts (Sheikh *et al.*, 2011). This framework was chosen because it applies to micro-level health systems, such as outreach clinics, and because it recognizes health systems as open, dynamic and fundamentally driven by human actions, beliefs and norms. It also provided a foundation for examining the influence of context on service responsiveness.

Throughout this analysis, detailed notes were recorded by the lead researcher to inform the interpretation of results. These included decisions made in the coding of transcripts, thoughts on emerging patterns and reflections on possible biases. Additionally, results were discussed among the researchers following each step of the analysis to address assumptions. The findings that emerged from the analysis were also validated through discussions with the NGO team that supported the integration of FP services in Malawi to enhance the trustworthiness of the analysis. Once the analysis was completed, example quotes were extracted for illustrative purposes, and the standards for reporting qualitative research checklist was used to improve the quality of reporting (O’Brien *et al.*, 2014).

Furthermore, quantitative data from the clinic audits were double entered from paper forms into EpiData and exported into STATA 16 to generate descriptive statistics summarizing the clinics’ characteristics. These are reported here along with a description of the respondents’ characteristics and a detailed explanation of the factors found to influence service responsiveness.

## Results

Overall, 23 clients and 10 HSAs who provided FP services across six clinics were interviewed. The clients interviewed had an average age of 23.5 years (ranging from 18 to 39), and most had one or two children (with older clients having up to six children). These clients included a mix of new and repeat contraceptive users, and almost all sought injectable contraceptives. Clients with one or two children reported using contraceptives for child spacing purposes, whereas clients with more than two children reported wanting to avoid additional pregnancies.

Of the six clinics selected for the SSIs, on the day of the interviews, four had a shelter and seating, one had multiple rooms, five met staffing standards (at least four HSAs) and five were staffed by HSAs who received FP training within the previous two years. Injectables were available in all clinics. However, in some clinics, demand exceeded available stocks, resulting in a few clients receiving pills or condoms as a stopgap. The average age of the 10 providers interviewed in these clinics was 39.3 years (ranging from 32 to 49). Among these providers, eight were male, and some lived in or around the communities they served whilst others resided further away.

**Figure 1.**
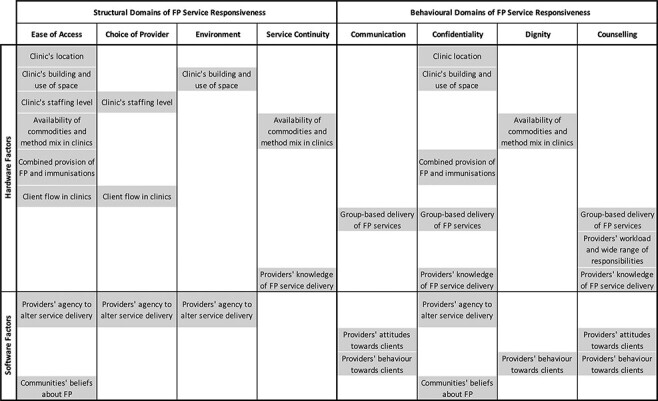
Factors influencing the responsiveness of integrated FP services in routine outreach clinics

In their accounts of the integrated FP services, respondents mentioned a total of 13 factors that they believed influenced service responsiveness. Among these factors, nine were hardware elements of the health system and four were software elements. As illustrated in Figure 1, almost all factors were perceived to influence service responsiveness via multiple domains.

### Hardware factors

#### The clinic’s location

According to both types of respondents (clients and providers), the clinic’s location influenced the responsiveness experienced by clients. Most clients believed that the clinics were well located because, by being community-based, they were closer than the nearest health facilities, which improved clients’ access to FP services. However, clients and providers noted that the providers struggled to reach the more remote clinics on time, which prolonged clients’ wait time for services. Additionally, the confidentiality offered to clients was believed to be undermined when clinics were located near main roads because community members could observe women seeking services.

*I think we have been respected because in the past we used to walk a very long distance to the clinic before this clinic was set up. It was very far* (Client_01).

*Contraception is very significant in such remote areas. For instance, in the first-place people were travelling long distances to access family planning methods…But now, people can access family planning methods in this place, so I think it is a great achievement that we have minimized the distance people have to travel* (Provider_09).

#### The clinic’s building

The delivery of services in an open space or in a building not suited to FP services (e.g. school or church) was cited as problematic. Both types of respondents felt strongly that a building with multiple rooms dedicated to the exclusive use of the clinic was crucial to avoid being seen or overheard whilst seeking FP services. However, opinions between clients and providers differed as to the importance of the clinic environment. On one hand, clients felt the clinic environment was the most important domain of responsiveness, and they believed an inappropriate environment raised doubts about the quality of the clinic’s services. On the other hand, providers believed that despite hindering service responsiveness, the environment was least important to clients and that clients would seek services in outreach clinics regardless of the environment given the clinics’ proximity to their homes.

*I don’t think there is order because there is no building; we are meeting under a tree. It shows a lack of development. Also, if there was a building, we would be accessing services without other people looking* (Client_01).

*…there is inadequate infrastructure and equipment, and this makes our job tough. You can see that we are actually using church buildings for shelter most of the time. So sometimes the church programmes overlap with ours and we have no choice but to go outside and conduct our clinic there. We do not have our own shelter where we can be free to offer these services, that’s the main challenge that we face* (Provider_04).

#### The clinic’s staffing level

Providers believed that responsiveness was compromized by staff shortages in clinics, which they attributed to off-site trainings, competing tasks and a lack of commitment among providers. Specifically, understaffing was seen as undermining the standardized client flow that providers relied on to integrate the delivery of services and consequently slowing the provision of services and increasing clients’ wait time. Providers also reported that it was impossible to give clients the opportunity to choose a provider when staffing levels were low but that it was an option when clinics were properly staffed.

*I think we need more staff here so that we divide our tasks well. When this is done, we will assist them* [the clients] *quickly and they won’t get tired of waiting…Some HSAs do not show up…We sometimes behave as if we have just realised that we should go to an outreach clinic. So, we begin preparations late and arrive late at the outreach and we bring few supplies…* (Provider_06).

#### The availability of commodities

In addition to preventing clients’ access to the contraceptives they required, pervasive commodity shortages in clinics were perceived to subvert the dignity afforded to clients. This is because, according to both types of respondents, clients equated accessing the services they needed and the contraceptive method they preferred to being respected. Providers explained that commodity shortages in clinics were commonly due to supplies originating from a single health centre with limited stocks being shared between multiple clinics.

*I am only complaining that I have pills and not the injection that I wanted because it is not available…With the injection, I take it once in 3 months. However, with pills then I need to take them once every day. I need to learn how to do this every day. I am not used to that* (Client_18).

Clients and providers also believed that the mix of contraceptive methods available in clinics influenced the service continuity experienced by clients. That is, both types of respondents suggested that referrals to distant health centres could be reduced by expanding the services provided in clinics to include the administration of LARCs (e.g. implants). Providers felt that clinics should be staffed with a nurse capable of providing these contraceptives, and they stressed that referring clients to health centres for LARCs was futile as few women followed through with the referral, opting instead for the short-acting contraceptives available in the clinics.

*There are long term methods which are not provided at this clinic. Methods like implants should be integrated and HSAs should be trained so that when we provide pills and injectables women can also access implants…Sometimes women consider the distance it will take them to reach the health facility to access a long-term method and eventually they do not go* (Provider_02).

#### The combination of FP and childhood immunization services

The combined provision of services was also perceived to influence service responsiveness. Both types of respondents emphasized that the combined provision of FP and childhood immunizations improved access to FP services by creating the opportunity to seek several services at once and reducing the direct and indirect costs associated with multiple visits. Clients and providers also felt that it facilitated confidentiality by making it less obvious to passers-by which women were seeking FP services in the clinic.

*…initially women would acquire immunization only here and get contraceptives somewhere else that was far away. As a result, most women opted not to go there. Some even bought expired contraception. But now it’s good that we are providing these services simultaneously as such we have more women coming and benefiting* (Provider_10).

#### The flow of clients in the clinic

Clients and providers reported that the standardized client flow adopted in clinics resulted in long wait times for FP clients (particularly when client loads were high) as FP services were provided after growth monitoring and immunization services. Providers suggested that wait times could be reduced by altering the client flow. Proposed alterations included (1) using a single provider to administer contraceptives and immunizations to clients seeking both services and (2) prioritizing these clients ahead of others. Clients also felt that the standardized client flow in clinics limited their choice of provider as each HSA was assigned to a specific service.

*Maybe we can improve on time so that women don’t stay long periods when they come here. Those coming for immunization and family planning stay in the same queue, so maybe we can split them so that those coming for family planning are treated first* (Provider_07).

*There is only one person who gives the [injections] so there is no chance of choosing* (Client_03).

#### The group-based delivery of FP services

The group-based provision of FP counselling (a common provider-led deviation from the standard operating procedure) was perceived to negatively influence responsiveness. Several clients also reported having difficulty hearing the group health talk at the start of the clinic day, which providers acknowledged was an issue when client loads were high. Clients explained that asking questions about FP in a group setting was not appropriate and that FP counselling should be provided individually instead of in groups to conserve women’s confidentiality.

*We were being taught as a group…Most of us wanted to take the injection but were shy to do so because we were told as a group…I was embarrassed to go and get the injection because everyone would see me…I would have taken the injection but because it was difficult to do so, I have just taken pills…The injection is what I normally use but today I have taken the pills. But I really would like to use the implant, Jadelle* (Client_16).

#### The providers’ workload

A common view among providers was that their workload also influenced service responsiveness. Several providers reported feeling overwhelmed by their workload given the many responsibilities they held in the health centres, clinics and community. They believed that the wide range of responsibilities they were tasked with compromized the services they delivered, especially the FP counselling they provided to clients in clinics.

*The workload that we normally have. We have to go and work in the communities and then go back and work at the clinic too. Because we are overwhelmed, we don’t perform our best…I have tasks in my area as well as at the facility. I get so overwhelmed and sometimes I just perform the tasks to fulfil duty* (Provider_03).

#### The providers’ knowledge

Providers believed they possessed the knowledge needed to deliver confidential services to clients. However, they expressed a need for additional and refresher training to strengthen their capacity to provide FP counselling and to answer clients’ questions. They also felt they should be trained to provide LARCs to make these available in clinics and improve the service continuity afforded to clients. Clients, however, commented very little on the providers’ knowledge and its effect on service responsiveness.

*That confidentiality is there because when we were starting integration, we were told about that during training. We were told to emphasize that whatever happens at the outreach is purely confidential and should not be revealed to husbands at home in anyway* (Provider_07).

*Sometimes a new method is introduced, or new drugs are introduced and as a provider we are usually just told to start providing without proper training. When new things are coming in, it is important to train us so that we are able to answer the questions coming from clients* (Provider_02).

### Software factors

#### The providers’ agency

Clients and providers reported multiple instances where the providers’ agency, or capacity to act independently, had a positive influence on responsiveness. This included identifying HSAs to fill team absences to ensure clients could access services quickly and choose a provider, altering the client flow to overcome issues associated with the lack of appropriate shelter and improving the confidentiality of services. For example, providers reportedly improved the confidentiality of services by (1) bringing clients behind the clinic or away from others to counsel them privately, (2) prioritizing clients requiring additional privacy (e.g. unmarried women) and (3) delivering services after clinic hours or in their own homes.

*No there is no privacy here. We can all see what method the other is taking…We should have one on one sessions with the health workers…The health worker who was coming for the past few months would tell us that if we want family planning, we should meet him by ourselves and we would have a health talk. It was good because nobody knew about your method* (Client_16).

*Also, there are people who do not want others to see them accessing family planning services for fear that they will tell their husband about it. For such women, we wait for everyone to go home, and they are the last to receive a service. They receive the service when everyone is gone including the relations of their husband* (Provider_05).

#### The providers’ attitude toward clients

The providers’ attitude toward clients was also key to the responsiveness experienced by clients. Providers believed the clients’ limited educational opportunities and knowledge about health services and FP impeded the counselling they delivered. Specifically, the providers felt that they needed to adopt a suboptimal approach when communicating information to clients to build their understanding slowly over time.

*This place is under development. I think communicating with people must be done in steps. You are educated but they are not…Bearing in mind that it is not simple to change a person, I think we have to take them slowly up until they are able to learn things faster* (Provider_09).

Equally, the providers viewed some clients’ behaviour as problematic. For example, providers perceived clients who sought services elsewhere (particularly during the farming season) or delayed follow-up visits as destabilizing service continuity. Similarly, providers felt that clients who interrupted the flow of services in the clinic hindered access to services by increasing other clients’ wait time. Providers also believed that clients who reached the clinic late or did not move quickly through the clinic’s different stations posed a challenge, as they missed the group health talk and/or FP counselling and were subsequently more likely to adopt a contraceptive with little understanding.

*The challenges are there. In this area, people are very mobile so when they take a method this month, the next month they move to the farming lands. They end up missing their appointment dates. They come back to this area when the farming season is over* (Provider_02).

*Another challenge is that some women pass their clinic cards to their friends whilst they stay back at home to come later, this holds us up, as we end up having to wait for them before we can start our sessions* (Provider_04).

#### The providers’ behaviour towards clients

In general, clients reported feeling respected by providers whom they viewed as professional and helpful. However, some clients’ remarks revealed that the providers’ attitudes towards clients sometimes resulted in disrespectful behaviour. Clients felt this undermined the dignity and counselling they experienced. And although providers mostly viewed their own behaviour towards clients positively, they acknowledged that clinic utilization rates would likely improve if they adopted a better attitude, greeted clients carefully and communicated more respectfully.

*…there are some* [clients] *that are shouted at for coming on a wrong date. The* [provider] *shouts and blames them for not checking their date properly in their book. I feel this is very disrespectful since most of the women are illiterate and do not know how to read. They are supposed to tell them in a polite manner because even if they are illiterate, they are still wise on other things* (Client_18).

#### The communities’ beliefs about FP

Finally, a common view among both types of respondents was that FP was generally perceived favourably by the communities in which the clinics operated. Clients and providers mentioned that community members believed FP was helpful to prevent unplanned pregnancies, to free up women’s time for other tasks and to limit population growth in a context of depleting natural resources and rising poverty. This favourable outlook was believed to aid clients’ access to FP services. However, both types of respondents also said FP was not openly discussed in communities because it was viewed as a private matter and that some women feared being bewitched for using contraceptives, which put pressure on providers to ensure the confidentiality of FP services. Also, most clients reported feeling supported by their husbands to use contraceptives. However, several clients mentioned that not all husbands were equally supportive, which was perceived to hinder women’s access to services and drive the need for confidentiality. The lack of support among some husbands was believed to be due to misconceptions about contraceptive side effects, such as infertility, reduced sex drive, erectile dysfunction and women no longer ‘being sweet in bed’. Providers highlighted that these misconceptions were in part the result of FP services not reaching men in the communities.

*People are afraid to disclose when they have accessed a family planning method because others will perform some magic on them causing them to have prolonged menses* (Client_12).

*I have been visited by such women* [who seek FP services in secret against their husband’s wishes] *because people are cognizant of the advantages of a small family. Many people in this area suffer from famine so they struggle to make ends meet. This can be exacerbated by having large families with four or more children* (Provider_09).

## Discussion

This study set out to identify the factors influencing the responsiveness of FP services that were integrated with childhood immunizations in routine outreach clinics by exploring the perceptions and experiences of clients and FP providers. In general, clients’ perceptions aligned with those of their FP providers. However, a notable exception was their contradicting opinions on the importance of the clinic environment. A possible explanation for this difference was that the providers overestimated the value of the improved access afforded to clients through the outreach nature of the clinics and consequently failed to recognize how important the environment was to their clients. Interestingly, no noteworthy differences were found among the views of respondents across clinic sites.

In all, nine hardware and four software factors were found to influence the eight domains of responsiveness studied. Taken together, these factors highlight that, in the studied clinics, responsiveness was a product of not only the organizational arrangement of resources but also the process involved in the provision of services and of the characteristics and behaviours of the actors interacting at the point of care. This corroborates Mirzoev and Kane’s conceptualization of responsiveness, which places the interaction between clients and their service providers at its centre (Mirzoev and Kane, 2017).

Among the hardware factors identified in this study, the clinic’s (1) inappropriate building and use of space, (2) group-based delivery of FP services, (3) staffing shortages and (4) lack of commodities were all perceived to negatively affect service responsiveness. These findings mirror the views expressed by clients of integrated FP and childhood immunization services in Benin, India, Liberia and in the Dowa and Ntchisi districts of Malawi (FHI 360, 2012; Cooper *et al.*, 2015; 2020; Nelson *et al.*, 2019; Erhardt-Ohren *et al.*, 2020). Of note, the absence of a dedicated and private space for the provision of confidential and dignified FP services was found to be especially detrimental to the responsiveness experienced by clients. This highlights the importance of identifying a fixed space that is appropriate for the delivery of FP services when these are integrated with childhood immunizations through community outreach platforms. Conversely, the combined provision of FP and childhood immunization services was mostly viewed by respondents to have a positive effect on service responsiveness. This is likely because the combination of these two services helped enhance the ease of access and confidentiality afforded to clients in a community context where health services were hard to reach and where myths and misconceptions about contraceptives rendered confidentiality paramount. These results are consistent with research from rural Liberia in which health facility clients who received bidirectional referrals between FP and childhood immunizations reported appreciating the chance to receive information about a service they did not originally intend to seek and the opportunity to access two services on the same day (Nelson *et al.*, 2019).

In contrast, the effect of software factors on service responsiveness was generally viewed more favourably. Among these, the providers’ agency emerged as a critical factor and was perceived to influence half of the responsiveness domains. That is, the providers’ use of their agency to overcome hardware shortfalls by altering the delivery of services was perceived to improve the ease of access, choice of provider, environment and confidentiality experienced by clients. This finding contributes to the emerging recognition that providers can offset the effect of some hardware deficiencies when services are integrated if they are afforded sufficient flexibility to make independent decisions (Mayhew *et al.*, 2017; 2020). However, these responses to hardware deficiencies are less likely to be effective when the hardware in question relates to the providers themselves (e.g. staffing shortages or knowledge gaps). Also, it is worth noting that despite this positive use of agency, some decisions made by providers were perceived to have the opposite effect on service responsiveness. For instance, the providers’ decision to deliver group-based counselling to streamline services instead of counselling clients individually per the standard operating procedures was believed to undermine the confidentiality, communication and counselling experienced by clients. Similar provider-led modifications were reported in Zambia where high demand for immunization visits limited the time allotted to the provision of FP services, which resulted in providers perceiving group-based services as more practical than individualized FP messaging (Vance *et al.*, 2014). Such trade-offs between the practicalities of delivering services and the responsiveness afforded to clients are to some extent unavoidable in practice (World Health Organization, 2000), particularly in resource-constrained settings. In fact, as established by Lipsky (1980) and further explained by Erasmus (2014), frontline health workers, at times referred to as ‘street-level bureaucrats’, commonly reinterpret policies and alter the delivery of services as a coping strategy to overcome resource constraints or heavy workloads by invoking their ‘discretionary power’. In doing so, these actors are likely to redefine or even contradict the aims of the policies and programmes they are tasked with implementing (Lipsky, 1980; Erasmus, 2014). Therefore, programme designers looking to improve the responsiveness of FP services that are integrated with childhood immunizations should not only create the conditions under which providers can apply their agency to pragmatically deliver services, but they should also provide the tools for them to adapt the provision of these services in a way that ensures responsiveness goals are achieved. To do so, it is essential for programme monitoring systems to capture clients’ experiences across all responsiveness domains and for feedback mechanisms to be implemented that enable providers to make data-driven decisions. However, it is equally vital to address broader structural determinants so that providers’ efforts are not dominated by attempts to mitigate the negative effects of hardware deficiencies (Topp and Sheikh, 2018). Additionally, examining provider behaviour and identifying behaviour change opportunities could also help improve service responsiveness as the findings from this study underline that providers’ attitudes towards clients can be biased and negatively impact the services they deliver (Breakthrough ACTION, 2020).

Overall, this study documented the effect of key factors on the responsiveness of FP services that are integrated with childhood immunizations and highlighted the critical influence of software factors. However, given the dynamic nature of health systems (de Savigny and Adam, 2009) and the complexity of service responsiveness, understanding the interrelationships between hardware and software factors represents an important next step to inform the design and delivery of responsive FP services that are integrated with childhood immunizations.

## Limitations

The findings from this study are somewhat limited by the small number of interviews. However, this is less concerning as the respondents volunteered a substantial amount and depth of information during the interviews, and the data captured across clinics indicated saturation was achieved. Also, by using a convenience sampling strategy instead of a purposive or systematic approach to recruit clients into the study, it is possible that important perspectives were missed. For example, a different sampling strategy could have provided the opportunity to interview clients who were interested in, or normally used, contraceptives other than injectables. Additionally, interviews with women opting not to seek clinic services and actors operating beyond the boundaries of the clinics could have provided additional insights, including the effect of upstream factors on service responsiveness. Further studies should aim to capture these complementary perspectives to enrich the findings reported here.

## Conclusions

The study investigated the factors influencing the responsiveness of FP services that were integrated with childhood immunizations in routine outreach clinics by examining the perceptions of clients and providers. Findings revealed that hardware factors, including the non-static nature of the clinics characterized by the absence of a dedicated and private space for the delivery of services, undermined the responsiveness experienced by clients. However, crucially, providers can mitigate the negative effect of such hardware deficiencies by applying their agency to alter service delivery. Consideration of software elements in the design and delivery of integrated FP services is therefore critical to optimize the responsiveness of these services.

## Data Availability

The datasets used and/or analysed in the current study are available from the corresponding author upon reasonable request.

## List of abbreviations

FP: family planning, HSA: health surveillance assistant, LARC: long-acting reversible contraceptive, NGO: non-governmental organization, SSI: semi-structured interview.

## Reflexivity statement

The authors include seven females and one male and span multiple levels of seniority. Three of the authors specialize in the delivery of public health interventions in Malawi, one is a family planning and reproductive health expert and the other four are researchers based in the UK with expertise in the evaluation of complex health interventions and quality of care. All authors have extensive experience conducting qualitative fieldwork in Malawi and other countries in sub-Saharan Africa.
